# A Comprehensive Review of High Burn-Up Structure Formation in UO_2_: Mechanisms, Interactions, and Future Directions

**DOI:** 10.3390/nano15050325

**Published:** 2025-02-20

**Authors:** Zhenhong Ge, Dong Yan, Penghui Lei, Di Yun

**Affiliations:** School of Nuclear Science and Technology, Xi’an Jiaotong University, Xi’an 710049, China; gezhenhong@stu.xjtu.edu.cn (Z.G.); flavia990331@stu.xjtu.edu.cn (D.Y.)

**Keywords:** uranium dioxide, high burn-up structure, recrystallization, polygonization

## Abstract

In the rim zone of UO_2_ nuclear fuel pellets, high burn-up and low temperatures drive changes in the microstructure, leading to the formation of high burn-up structures (HBS). This review focuses on the formation of HBS, beginning with a description of the two contentious mechanisms—recrystallization and polygonization—that are believed to be the primary controlling factors. We discuss experimental and simulation studies that support both mechanisms, emphasizing that although each mechanism can explain certain aspects of HBS formation, neither recrystallization nor polygonization alone is sufficient to fully explain the observed phenomena. Furthermore, we emphasize the intrinsic relationship between these two mechanisms, suggesting that they represent different manifestations of the same underlying process under varying conditions, and we reference relevant studies that support this perspective. Lastly, we underline the significance of investigating the formation processes of HBS and provide an outlook on future research directions based on the current state of knowledge.

## 1. Introduction

Due to the neutron energy spectrum configuration in pressurized water reactors (PWRs), the intense radiation self-shielding effect can cause the UO_2_ fuel pellet near the edge to experience local burn-up exceeding the average burn-up by more than double. When the local burn-up reaches around 50 GWd/tHM, a noticeable grain refinement process begins, resulting in a new fuel morphology. By the time the fuel burn-up reaches approximately 75 GWd/tHM, this transformation is nearly complete. This newly formed structure is referred to as HBS, also known as the rim structure, rim effect, or described as “cauliflower-like” [1] based on its morphology.

It is generally accepted that HBS exhibit the following characteristics:

(1) The original grains, typically measuring around 10 μm in the fresh fuels, are subdivided into 10^4^–10^5^ new grains, each measuring 100–300 nm;
(2) The appearance of micron-sized intergranular fission gas pores, indicating a larger porosity in the HBS;
(3) The depletion of Xe and dislocations in the newly formed fine-grained matrix.

The HBS occupy only a thin region at the radial edge of the fuel and expand inward radially as burn-up increases. Some researchers [2] believe that under suitable conditions, this expansion can extend deeply into the fuel. Moreover, the formation of such HBS has been observed not only in UO_2_ fuel but also in Pu-rich mixed U-Pu fuels (MOX) and U-Mo fuels [3]. Similar grain refinement phenomena, potentially indicative of HBS formation, have also been observed in carbide [4] and silicide fuels [5].

As early as the 1950s, localized restructuring phenomena were noted in naval reactors [6] and were observed in commercial reactors in the early 1960s [7]. However, with the ongoing development of commercial reactors aimed at alleviating the rapid increase of spent fuel and enhancing economic efficiency through higher operational burn-up, the newly formed HBS have raised concerns about the safety implications of increased burn-up, especially after studies indicating degradation in thermal conductivity [8] and mechanical properties [9] associated with HBS.

Thus, the importance of measuring and modeling HBS to assess their impact during operation has become increasingly prominent. This raises the question of how HBS are formed, and the implications of this formation process are unavoidable for common burn-up levels in commercial PWRs. In the following sections, we will discuss research on the formation of HBS and present some insights.

## 2. The Formation Mechanism of HBS: An Unresolved Issue

Due to the complexities of nuclear fuel systems and the numerous influencing factors, coupled with the harsh service environments and objective limitations of post-processing, the application of characterization techniques is significantly restricted. These combined factors result in a lack of systematic and comprehensive scientific descriptions of the formation of HBS. Existing experimental results can only focus on microstructural morphology and elemental distribution in localized areas at specific burn-ups, without capturing the continuous changes in microstructural characteristics at the same location under varying burn-up conditions. The prolonged cooling time after removal from the reactor introduces errors, which notably affect the observation of the microstructural features. Furthermore, experimental constraints in early characterization further limit the observation of this process.

**Figure 1:**
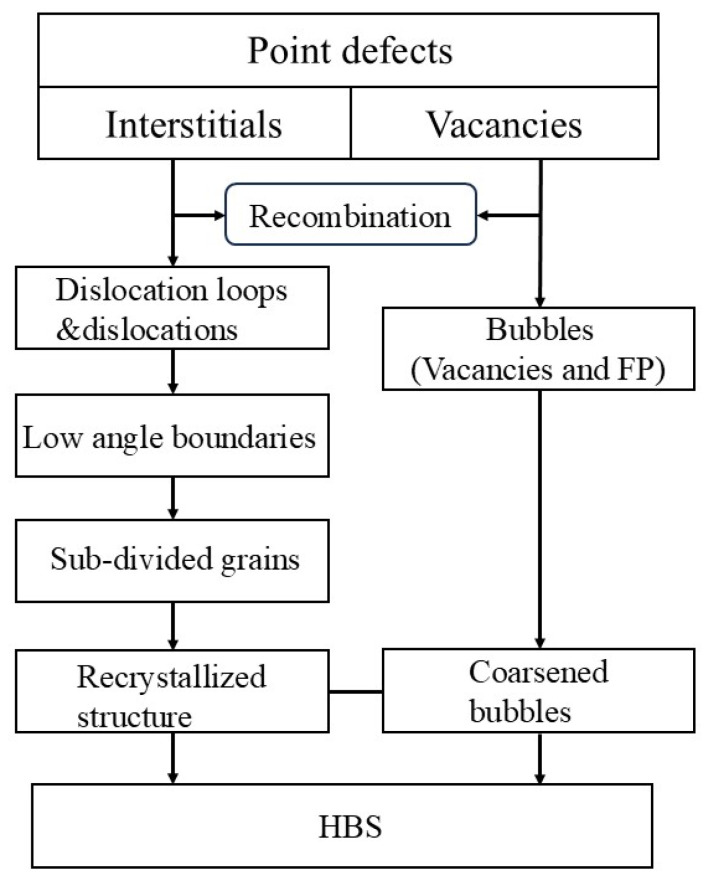
The recrystallization mechanism proposed by Nogita and Une et al. [10].

**Figure 2:**
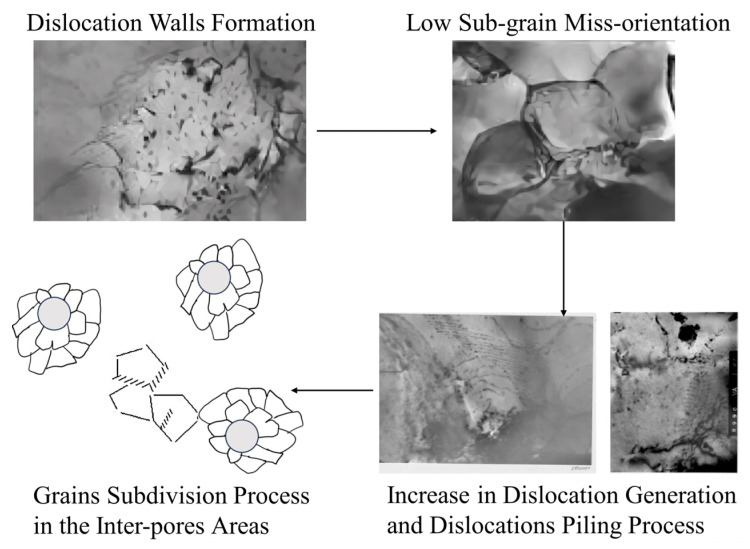
The polygonization mechanism proposed by Baron et al. [2].

There is a widespread debate regarding the mechanisms behind HBS formation, with two prominent perspectives: one posits that HBS arise from the formation and growth of subgrains into recrystallization nuclei, followed by the growth of recrystallized grains, representing a recrystallization mechanism, as shown in Figure 1. The other suggests that they result from the entanglement of dislocations forming dislocation walls, which then rearrange, leading to the subdivision of original grains, representing a polygonization mechanism, as shown in Figure 2. Both mechanisms are supported by relevant experimental evidence, such as the presence of numerous nano-grains with high-angle boundaries, generally regarded as features of recrystallization, observed in edge-region EPMA characterizations, as well as the newly formed relatively large grains exhibiting low-angle boundaries, which could only arise from polygonization.

Discussions based on these mechanisms continue to emerge, along with preliminary explorations of evolutionary process models and their impacts based on experimental characterizations. The establishment of these models also reflects the ongoing debate surrounding these mechanisms. Since the review by Rondinella et al. [5] has already provided a thorough summary of earlier literature, this overview will primarily focus on research related to HBS conducted after the year 2000.

### 2.1. Observation from the Experimental Perspective

Nogita and Une [10,11] proposed that at lower burn-up (44 GWd/tU), the tangled dislocation network with low-angle grain boundaries is formed by the inhomogeneous accumulation of dislocations. In contrast, for higher burn-up fuels, the energy stored in the matrix is released, resulting in the original grains being subdivided into high-angle grain boundary grains of 20–30 nm size, which serve as the nuclei for subsequent irradiation-induced recrystallization. The strain energy in the matrix, driven by the accumulation of irradiation defects, facilitates recrystallization, and past research work indicates that temperature has a significant impact on this process. They also noted that the recrystallization process eliminates intragranular bubbles and dislocations, asserting that recrystallized grains do not undergo secondary recrystallization. Furthermore, the relationship between dislocation density and burn-up was summarized (although the temperature effect is relatively limited, especially within the lower temperature range at the periphery): log(*ρ_d_*) = 2.2 × 10^−2^*β* + 13.8. This relationship is widely utilized in phase-field simulations to change the free energy. Une further noted in experiments [12] with large grain-sized UO_2_ fuel that larger grain sizes significantly enhance resistance to HBS. It was also observed that high-density dislocations and subdivided grains first appear at the grain boundary locations, providing additional support for the proposed HBS formation mechanism.

Spino et al. [13] used a specially developed micro X-ray diffraction technique to measure the radial variations in lattice parameters and peak widths in two high burn-up UO_2_ fuels (67 and 80 GWd/tM). They found that α-damage decreases both toward the hotter center and the cooler edge of the pellet. The reduction toward the center was attributed to thermal annealing, while the reduction toward the pellet edge was linked to the formation of the HBS. They also noted that the formation of the HBS requires significant lattice damage and the accumulation of xenon gas atoms trapped within the matrix.

Noirot et al. [14] concluded from experimental observations that the formation of HBS is driven by fission products, rather than fission itself. They suggested that there is a threshold temperature, above which subgrains do not form. Their observations of samples at 45 GWd/tU revealed that HBS appear within grains, and the impact of grain size on grain boundaries may have a limited role in inhibiting HBS formation. Based on the experimental evidence, they proposed that HBS initiation is primarily triggered by the solid solution of trivalent rare earths and plutonium, rather than by gaseous, metallic, or volatile fission products. However, they also noted that subsequent changes involving bubble formation indicate that these other fission products contribute to the further development of HBS. Further examination [15] of the M5-clad fuel irradiated to 83 GWd/tU indicated that the fuel porosity gradually increased between fuel radial locations of 0.43R and 1R, where R denotes the fuel radius, with only a limited increase in the range of 0.43R to 0.57R. It is noteworthy that the large, irregular (non-spherical and far from lens-shaped) bubbles formed at the center of the fuel retained most of the unreleased fission gases, with the majority of these bubbles located at the boundary [16].

Baron et al. [2] proposed that the development of the HBS occurs in three sequential stages: the construction of a periodic dislocation network, the loss in EPMA gas detection, and the coalescence and growth of intragranular nanoscale bubbles followed by grain refinement. Based on experimental observations, with burn-ups above 55 GWd/tU, they suggested that this grain refinement is predominantly driven by the polygonization mechanism, which contradicts the perspective of Nogita and Une et al. [10,11]. Additionally, they speculated that the propagation of grain subdivision may be caused by the punching effect due to pressurized pores or the high stress in the regions between pores.

T. Sonoda et al. [17] characterized 16 fuel samples irradiated at temperatures ranging from 400 °C to 1300 °C, with burn-ups of 36 to 96 GWd/tU. Building upon the aforementioned experiments, they further expanded the temperature and burn-up ranges in their study and suggested that both temperature and burn-up play a critical role in the formation of HBS. They observed that the HBS transformation threshold occurred between 55 and 82 GWd/tU, with a temperature threshold of 1100 ± 100 °C. TEM analysis revealed that most subgrain boundaries were of low-angle type, and 0.5–2 µm polygonal grains were also present, strongly supporting the polygonization mechanism. In high-temperature irradiated samples, dislocations appeared to be pinned by fission products, indicating a possible pinning effect. They further conducted ion irradiation experiments [18] on UO_2_ using 100 MeV Zr^10+^ and 210 MeV Xe^14+^ ions to study the properties of ion tracks and the microstructural evolution under ion track accumulation. The experimental results indicated that ion irradiation leads to shape changes in fabricated pores (from spherical to elliptical) and dislocation generation. The significant changes in both the surface morphology and internal structure of UO_2_ suggested that the overlapping of ion tracks enhances the diffusion of point defects and dislocations, facilitating subgrain formation at relatively low temperatures. The study also suggested that subgrain formation could occur independently of fission product presence.

In the New Cross-Over project (NXO), Kinoshita et al. [19,20] used Xe ion implantation in CeO_2_, which has a similar fluorite structure to UO_2_, to recreate subgrain structures analogous to those found in high burn-up fuels. Through TEM and synchrotron radiation XRD, they determined that oxygen plays a crucial role in the reorganization and recovery of nanoscale crystals. Additionally, computational results from first-principles calculations [19,21], molecular dynamics [20], and accelerated molecular dynamics [19,22,23] further supported the idea that oxygen atom clusters and the resulting stable structures may play a significant role in the formation of HBS.

Miao et al. [24] irradiated microcrystalline UO_2_, produced by plasma sintering, with 84 MeV ions at 350 °C to a dose of 1357 dpa, discovering the presence of nano-grains and pores characteristic of HBS. They conducted a quantitative study on the sizes and number densities of the formed bubbles as well as the grain size distribution, analyzing the misorientation of the grain boundaries. Their findings provide partial support for the perspectives proposed by Baron et al. [2] regarding the formation process of HBS. It was found that low-angle grain boundaries predominated (83%), leading to the conclusion that the formation mechanism of HBS is a polygonization process driven by radiation-induced dislocations associated with pipe and grain boundary diffusion of Xe and vacancies.

Baranov et al. [25] conducted ion irradiation of SIMFUEL fuel in the energy range of 20 keV (He^+^) to 320 keV (Xe^16+^) and 1–90 MeV (Xe^24+^). They observed grain refinement only under high-energy ion irradiation. They posited that, for low-energy ions, the size of the coherent scattering regions decreases with increasing dislocation density, which limits dislocation motion and subsequently restricts the formation of subgrains. In contrast, high-energy ions, upon reaching critical conditions, increase the size of the coherent scattering regions, allowing for dislocation rearrangement and enhancing dislocation diffusion creep, thereby promoting grain refinement based on the polygonization mechanism. They also noted that dispersed particles, represented by small bubbles, can hinder dislocation movement, further limiting grain refinement.

Gerczak et al. [26] analyzed the nearly complete cross-section of high burn-up commercial fuel with local burn-ups of 60 to 184 GWd/tU. The fuel experiences significant microstructural variations from the fuel-cladding interface to the fuel pellet center, influenced jointly by temperature and local burn-up, which aligns with the structural changes initially observed by Spino et al. [13]. By examining grain boundary misorientation and size, they identified a transition region near fully developed HBS and suggested that the grain refinement is primarily driven by polygonization mechanisms due to the formation of low-angle grain boundaries. They also speculated that the disappearance of low-angle boundary features within the region transitioning to HBS is caused by creep deformation in the pellet. Cappia et al. [27] irradiated UO_2_ samples with original grain sizes of 7.2/10 μm at 623/723 K using 84 MeV Xe^26+^ ions to doses of 1357/1493 dpa. They also observed that low-angle grain boundaries dominated the subgrain boundaries. However, in the regions where grain refinement occurred, no bubbles were found, leading them to conclude that the presence of large bubbles is not a necessary condition for grain refinement. This contradicts the viewpoint of Baron et al. [2].

Hiernaut’s annealing experiments [28] on high burn-up samples with a local burn-up of 220 ± 20 MWd/kgHM showed that a significant portion of the fission gases remains trapped within the HBS. The pressure within the HBS pores can reach up to 30 MPa, causing grains to fracture along the original grain boundaries. This indicates that overpressure may be a contributing factor in the formation of the HBS.

Xiao et al. [29] irradiated samples under conditions of linear power ranging from 260 to 300 W/cm and temperatures between 300 and 400 °C, achieving burn-up depths of 48–120 GWd/tU. High-resolution scanning electron microscopy (SEM) was used to observe fuel samples with varying local burn-up (at different radial positions) within the 48–120 GWd/tU range. Observations of low burn-up samples revealed the formation of subgrains with low-angle boundaries away from coarsening bubbles, which is consistent with the findings of Baron et al. [2] on grain refinement. As burn-up increased, small grains with high-angle grain boundaries appeared around the bubbles, alongside the formation of new low-angle subgrains. In the highest burn-up samples, a greater number of high-angle grain boundaries surrounded the coarsening bubbles. Based on these findings, they concluded that both polygonization and recrystallization are mechanisms contributing to the formation of HBS.

Barker et al. [30] examined approximately 30 AGR samples exhibiting HBS after irradiation using SEM and light optical microscopy (LOM), along with similarly sized AGR fuel samples without HBS. They observed that the minimum burn-up for the emergence of HBS was 27 GWd/tU, suggesting that this earlier threshold burn-up may be influenced by the moderator. They also noted that the early stages of HBS formation seem to initiate within the grains, indirectly supporting the polygonization mechanism.

Cappia et al. [31] conducted a study on the microstructural features and volatile fission products of high burn-up samples with local burn-up reaching 76 GWd/tHM across different radial positions. Experimental observations revealed that irradiation introduced a significant number of defect damages (this observation is consistent with the findings of T. Wiss et al. [32]), resulting in the formation of a dislocation network. From a microstructural perspective, three distinct regions were identified along the radial position:

1. In the central region, noticeable polygonization occurred, resulting in subgrain domains separated by low-angle grain boundaries;
2. In the region corresponding to a relative radius of 0.55 to 0.8, there were no clear features of grain subdivision, lower porosity compared to the central area, and a high retention of nano-sized bubbles in the matrix;
3. At the outer region, near a relative radius of 0.98, clear HBS characteristics were observed.

Zacharie-Aubrun I et al. [33] characterized standard and Cr-doped UO_2_ samples within a burn-up range of 35–73 GWd/tU using EBSD techniques. They observed distinct HBS at the fuel edges, which was independent of the initial microstructure. For all types of samples, when the burn-up exceeded 61 GWd/tU, a restructuring phenomenon was evident in the central region, believed to be related to the formation of bubbles. Furthermore, they noted that this restructuring intensified with increasing burn-up, and the orientation of the grains formed in the restructuring area was similar to that of the original grains.

Based on the aforementioned experimental studies, the formation conditions of HBS in UO_2_, including temperature, burn-up threshold, and the original grain size—an important influencing factor identified in numerous experiments—along with the proposed formation mechanisms and fuel types, are summarized in Table 1. Here, a single temperature value corresponds to the burn-up threshold for the formation of the HBS at that temperature.

### 2.2. Insights Derived from Numerical Simulations

In the simulation study of HBS formation, an attempt was first made to derive the required kinetic parameters for the evolution of the kinetic equations by combining experimental data and theoretical assumptions. The evolution of point defects, dislocations, bubbles, and other defects with burn-up at different temperatures was investigated. For instance, in the treatment of fission gas bubbles, the following approach is commonly adopted [34]:

(1) $$\frac{dC_{\alpha ,i}}{dt}\;=\;-\;a_{\alpha ,i}C_{\alpha ,i}^{2}\;-\;b_{\alpha ,i}C_{\alpha ,i}\;+\;e_{\alpha ,i}$$

where *i* represents the size class of the bubble, which is distinguished according to the number of gas atoms contained in a bubble; *α* represents the location of the bubble: lattice, dislocation, grain face, and grain edge. *C_α_*_,*i*_ represents the number density of the *i*-th size class of bubbles at the *α* position (when *i* = 1, the number density corresponding to the fission gas atoms is changed to *C_g_*); *a_α_*_,*i*,_ *b_α_*_,*i*,_ *e_α_*_,*i*,_ are the rate coefficients representing different fission gas behaviors affecting the bubble number density, respectively. Based on these computational results mentioned before, a relationship between the computed values and the experimentally observed threshold for HBS formation was established. This criterion allows for effective simulation of the restructuring process.

Martín Lemes et al. [35] observed a significant change in behavior around 100 MWd/kgU based on experimental data [36,37] regarding porosity and pore number density changed with burn-up: the rate of increase in porosity slowed down, while the pore number density reached a maximum before declining. Consequently, high burn-up was categorized into two cases: 60–100 MWd/kgU and greater than 100 MWd/kgU (defined by Romano A [37] as ultra HBS. Using empirical hydrostatic pressure equations that vary with burn-up and a modified Van der Waals equation, along with established empirical equations for porosity and pore number density, they studied the fission gas overpressure and the retention of fission gases in the matrix and pores under the two cases separately. The results compared well with existing published data. This threshold is confirmed by the pore size study of fuels with a burn-up of 80–200 MWd/kgU by Cappia et al. [38].

Rest and Hofman analyzed grain refinement based on the principles of metal recrystallization [39,40]. Their theory posits that during irradiation, cellular dislocation structures form at an early stage. Impurities generated during fission subsequently diffuse to the void dislocation structures as vacancy-impurity complexes, thereby hindering dislocation motion across the walls. The impurity-free outer walls further undergo subgrain coalescence. The core hypothesis for the recrystallization process is to control the concentration of unit volume nuclei through kinetic equations. As the fission process progresses, energy becomes increasingly concentrated in fewer nuclei. Grain refinement occurs when the creation of grain boundary surfaces is offset by the creation of strain-free volume, resulting in a net decrease in the material’s free energy.

The model subsequently considers the effects of impurity pinning and fission gas bubbles on the recrystallization nuclei [41]. Furthermore, based on observations and modeling of the U-Si compound system [42,43], the authors incorporate the amorphous clusters generated by collision cascades and the centers of compression produced by “shock waves” into the model as influencing factors for nuclei control. Finally, using the same theory, they extend this recrystallization simulation to U-xMo fuel with similar phenomena [44], explaining the depletion of Xe, pore formation [45], and swelling [46] effects influenced by the recrystallization process. Rest also noted that the model is supported by the experimental observations of submicron recrystallized grains in 100 GWd/tU fuel made by Thomas et al. [47] and Nogita et al. [10].

Veshchunov et al. [48] developed and implemented a model for dislocation generation and evolution under irradiation conditions in the MFPR framework, combining it with a set of equations for point defect evolution and their interactions with bubbles. This model was validated against the experimental data of Nogita [11]. Formation and growth of intragranular fission gas bubbles in UO_2_ fuels with burn-up of 6–83 GWd/tU provides a self-consistent consideration of the entire system in irradiated fuel, including point defects (vacancies, interstitial atoms, and gas atoms) as well as extended defects (bubbles, dislocations, vacancy loops, and pores). The model predicts a significant reduction in dislocation density at temperatures between 1300 K and 1400 K. Furthermore, at burn-ups of at least 100 GWd/tU, dislocation density does not reach the threshold, preventing grain restructuring. This is consistent with the observations of Kinoshita et al. [20]. Based on this, it is further suggested that the dislocation density may be a key factor influencing the temperature threshold for restructuring of HBS.

Xiao et al. [49] developed a model referencing the mechanisms proposed by Rest [39,40], which incorporates dislocation evolution under irradiation and the successive grain subdivision process as local burn-up increases. They hypothesize that the primary driving force for grain subdivision in the edge regions of high burn-up UO_2_ fuel matrices is the generation and accumulation of dislocation loops under irradiation. Based on this, they predicted the sizes of subdivided grains at different burn-up levels (50–135 GWd/tU) and dislocation density (6 × 10^16^–2.7 × 10^17^ m^−2^), and the simulation results showed good agreement with the experimental data [25]. The calculation results for dislocation density in the temperature range of 900–1400 K indicate that the dislocation density of as-irradiated UO_2_ is highly insensitive to temperature changes and decreases fleetly within this range. It is further noted that grain refinement is likely to occur in the temperature range of 1300–1400 K only when the burn-up reaches at least 100 GWd/tU, which is consistent with experimental observations [17].

D. Pizzocri et al. [50] did not discuss the mechanisms of polygonization or recrystallization. Instead, they based their work on the concept of effective burn-up (which considers radiation damage accumulation only when the temperature is low enough to avoid thermal recovery of radiation damage) and measured grain sizes from experimental data. They directly fitted a first-order differential model to obtain the evolution equation for grain size as a function of burn-up, which aligns well with the experiments conducted by Ray [51] and Spino [36].

The phase field method, driven by the reduction of free energy, can simulate the microstructural evolution process at the mesoscopic scale, allowing for the study of microstructural morphology and the effects of external fields (such as thermal, mechanical, and irradiation). The evolution of HBS is described using a governing equation similar to the following form [52], which accounts for changes in energy:

(2) $$F\;=\;\int_{V}\left[f_{\mathrm{chem}}\;+\;f_{\mathrm{poly}}\;+\;f_{\mathrm{st}}\;+\;W\;g\left(\eta \right)\;+\;f_{\mathrm{grad}}\left(\eta ,\varphi \right)\right]dV$$

where *f_bulk_* represents the chemical free energy density, *f_poly_* is the polycrystalline energy density, *f_st_* is the stored strain energy density, and *f_grad_* is the gradient energy density, respectively. *g*(*η*) is the double well function and *W* is the potential height.

A modified Cahn–Hilliard equation is employed to describe the concentration changes of conservative field variables, such as vacancy and gas atom concentrations. For example, the governing equation for fission gas atom evolution is expressed as

(3) $$\frac{\partial c_{g}}{\partial t}\;=\;\nabla \;\cdot \;M_{\upsilon }\nabla \mu_{\upsilon }\;+\;P_{g}$$

where *M_g_* is the mobilities of Xe gas atom with the form and *P_g_* is the source term introducing gas atoms in the matrix phase.

The nucleation probability is calculated in a manner related to dislocation density (burn-up), thereby introducing nano-grains, which subsequently influence the free energy changes and simulate the evolution of the microstructure. Over the past decade, it has been widely applied in the field of microstructural evolution of nuclear fuel.

Abdoelatef et al. [53,54] established a phase field model on the MOOSE platform, considering interfacial energy at grain boundaries and bubble surfaces, dislocation-related strain energy, and the chemical energy of gas atoms to describe the formation and evolution of HBS. The simulation results indicate that dislocation density, grain boundary energy, bubble radius, and number density significantly influence the recrystallization fraction, grain count, and average grain size.

K. Ahmed et al. [55] addressed the recrystallization nucleation problem by considering simplified Langevin-type fluctuations in the evolution equations. They derived a critical dislocation density threshold for recrystallization: when the dislocation density exceeds this threshold, recrystallized grains grow; otherwise, they shrink. The evolution of dislocation density is also based on an empirical relationship [10] that depends solely on burn-up. The model further developed [56] has been shown to successfully simulate both homogeneous and heterogeneous nucleation of bubbles and subgrains. The computed subgrain size results align well with experimental findings [49] at 1200 K.

Amani Cheniour et al. [57] utilized the MARMOT based on the MOOSE platform and classical nucleation theory to simulate recrystallization at fixed core sizes under typical conditions of commercial light water reactor fuel and accelerated conditions at 650/800 °C and 40–60/40–90 MWd/kgU. The simulation results revealed significant differences in the variation of recrystallization fraction and starting point position, highlighting the notable impacts of power and temperature.

Permann et al. [58] introduced recrystallization grains using a random nucleation approach and established the concept of effective dislocation density based on the mechanism by which recrystallization eliminates dislocations. They used the formula proposed by Staker [59] to establish a relationship between the newly introduced grains and dislocation density, simulating the size and quantity of recrystallized grains based on the MOOSE platform.

Y. Jiang et al. [60] studied the impact of recrystallization grains on bubbles in UMo through random nucleation. The increase in grain boundary area fraction from recrystallization accelerated the formation and growth of bubbles. They further applied the explicit dynamic nucleation theory proposed by R. Ding [61] to introduce a nucleation rate for HBS of UO_2_ [52], calculating the nucleation rate per unit grain boundary area in the Arrhenius form. This successfully described the three-stage variation in porosity under synchronous bubble evolution conditions at 673 K and burn-ups ranging from 0 to 144 GWd/tU, aligning with experimental observations [26]. They also pointed out that the recrystallization process can promote bubble evolution, with the increased grain boundary area accelerating the nucleation of recrystallized grains and bubbles.

W. Jiang et al. [62] proposed a phase field model for quasi-brittle fracture in HBS with pressurized cracks under 700 K. It is suggested that the formation of HBS induced by the recrystallization mechanism affects the grain structure, thereby altering the crack propagation path and the morphology of the resulting fragments.

Baranov [63] established a dislocation dynamics model based on four premises: (P1) dislocations generation from radiation source are slow, (P2) because fresh fuel is brittle, dislocations are fixed after their generation by the radiation source, (P3) glid mobility is unaffected by irradiation, and (P4) short-term irradiation can be considered steady-state. The study investigated the distribution of dislocations and the effects of irradiation on dislocation motion. It was noted that, under the experimentally observed dislocation density and at a temperature of 573 K, interacting dislocations exhibit a periodic structural characteristic with spacings of 100–300 nm. This supports the view that HBS are induced by polygonization mechanisms and provides predictions for the minimum subgrain size produced by polygonization.

Jonnet [64,65,66] assumed that the formation of HBS is a polygonization process, where dislocations are essential for the formation of new subgrain boundaries. Therefore, his research model focuses on the role of dislocation-type defects—specifically, the nucleation, growth, and accumulation of interstitial-type dislocation loops formed by the necessary shear dislocation lines and the redistribution of point defects in the fuel. Based on this approach, he analyzed the theoretical stresses induced by the presence of various possible configurations of dislocations and emphasized the growth mechanisms of interstitial-type dislocation loops. This work provides theoretical support for further studies on the mechanisms of subgrain formation.

Based on the aforementioned numerical simulations, the formation conditions of HBS in UO_2_, including temperature, burn-up threshold, and the original grain size—an important influencing factor identified in numerous experiments—along with the proposed formation mechanisms, are summarized in Table 2. Here, a single temperature value corresponds to the burn-up threshold for the formation of the HBS at that temperature.

In summary, both experimental and simulation studies face difficulties in determining the precise temperature and burn-up values required for the formation of HBS, with these typically being presented as ranges or estimates. Due to the limitations of experimental setups, it is challenging to precisely capture the specific threshold for HBS formation, and the observed phenomena are constrained by the sample conditions. Furthermore, the parameterization in simulation studies is heavily dependent on the experimental characterization results. The debate between these two perspectives persists, influenced by varying fuel characterization outcomes and the limitations of the implementation methods.

## 3. Proposal of Hybrid Control Mechanism

### 3.1. Theoretical Basis

The above research all put forward their own views based on the observation of the actual experimental situation and explored the establishment of the model to a certain extent. However, the aforementioned studies generally consider HBS to be local phenomena that arise under specific temperature and burn-up conditions, predominantly governed by a single mechanism. From a holistic perspective on the fuel, one can argue that both mechanisms share commonalities or, further, that they represent different manifestations of a universal mechanism under varying conditions.

Based on existing experimental observations, the following hypothesis is proposed: from an energetic perspective, the influences of both mechanisms can be considered collectively. Liang et al. [67] proposed a relationship regarding the energy of deformed grains, suggesting that the energy accumulated due to dislocations is proportional to dislocation density and the shear modulus. Due to the influences of irradiation and fission products, dislocations continuously accumulate during the irradiation process and become pinned, resulting in a significant increase in energy within the system. Concurrently, influenced by fission gases or other volatile fission products, pores continuously form during the fuel operation, with their volume affected by local power and temperature. The recovery of dislocations is significantly temperature-dependent; as temperature increases, dislocation density substantially decreases.

As mentioned in Section 2.1, Nogita et al. [10] pointed out that as burn-up increases, the dislocation density gradually accumulates. The decline in dislocation density, thereby lowering the system’s energy, can only be achieved through recrystallization. In the central region of the fuel, although the temperature is higher, dislocation density is relatively low. However, from the modified Van der Waals equation, it can be inferred that higher temperatures lead to larger fission gas bubbles, which, on the one hand, adversely affect material properties and, on the other hand, pin the dislocations, further increasing the energy of the system. In the high-temperature region, the crystals rearrange dislocations to form low-angle grain boundaries, which leads to a configuration that lowers the internal energy of the system. However, when this energy has not reached the threshold necessary for the recrystallization process to occur, recrystallization cannot be initiated. This observation aligns with the previously mentioned polygonization theory.

For what is traditionally considered HBS, subgrain formation can, to some extent, be regarded as a precursor stage to the recrystallization process. Humphreys et al. [68] indicate that the nucleation stage of recrystallization occurs through the mechanism of subgrain growth, where recrystallization nucleation should take place in regions of high strain energy and high orientation gradients leading to discontinuous subgrain growth. Given that these conditions fall within the optimal temperature range for UO_2_ recrystallization (where reported grain refinement commonly occurs), coupled with significant burn-up differences due to radial power discrepancies (as indicated in research by D. Pizzocri [50]), the local high dislocation density achieved is insufficient to reduce system energy below the energy threshold, prompting a series of recrystallization processes.

Due to the significant reduction in grain size occurring concurrently with recrystallization, the migration of gas atoms and bubbles within grains toward the grain boundaries is markedly enhanced. This is facilitated both by the noticeably shortened diffusion paths and the effective trapping of these processes by the pores. Additionally, this process is accompanied by the transformation of original intragranular bubbles into intergranular bubbles (potentially captured by dislocation structures), further exacerbating this migration process. This leads to the observed phenomenon of intragranular Xe gas atom depletion in the matrix, further forming pores, which enhances local energy and accelerates the recrystallization process. Further observations [33] in the fuel center region support this notion. As the burn-up increases, larger pores gradually form within the center. The potential overpressure at these pore locations, combined with dislocation accumulation, significantly raises the local energy, thereby triggering a limited recrystallization process. This seems to provide theoretical support for Baron et al.’s [69] energy-threshold-based assessments.

For grains still near the edge but not exhibiting HBS, although they initially possess suitable temperatures for recrystallization, their lower burn-up leads to a relatively lower accumulated dislocation density, a considerable portion of which is reduced by subgrain formation. In the analysis of radial positions slightly greater than 0.5R, experimental observations suggest that this location corresponds with specific temperature conditions: Gerczak et al. [26] observed this at 0.63R (about 64 GWd/tU), Cappia et al. [31] at 0.6R (about 76 GWd/tU), and Zacharie-Aubrun I et al. [33] indicated it is just above 0.5R (about 61 GWd/tU). At this position, the temperature has departed from the optimal conditions for recrystallization. Additionally, as the temperature rises, dislocation recovery is enhanced, reducing dislocation density and thus diminishing the occurrence of recrystallization. On the other hand, with a relatively low temperature compared to higher temperatures where fission gas becomes quite mobile, the volume proportion of pores is limited, leading to a minimal pinning effect on dislocations. This keeps the system energy at a lower level, rendering dislocation rearrangement insufficient to drive subgrain formation compared to the central regions, resulting in most grains still exhibiting original grain morphology.

### 3.2. Existing Research Supports Hybrid Mechanism Control

**Figure 3:**
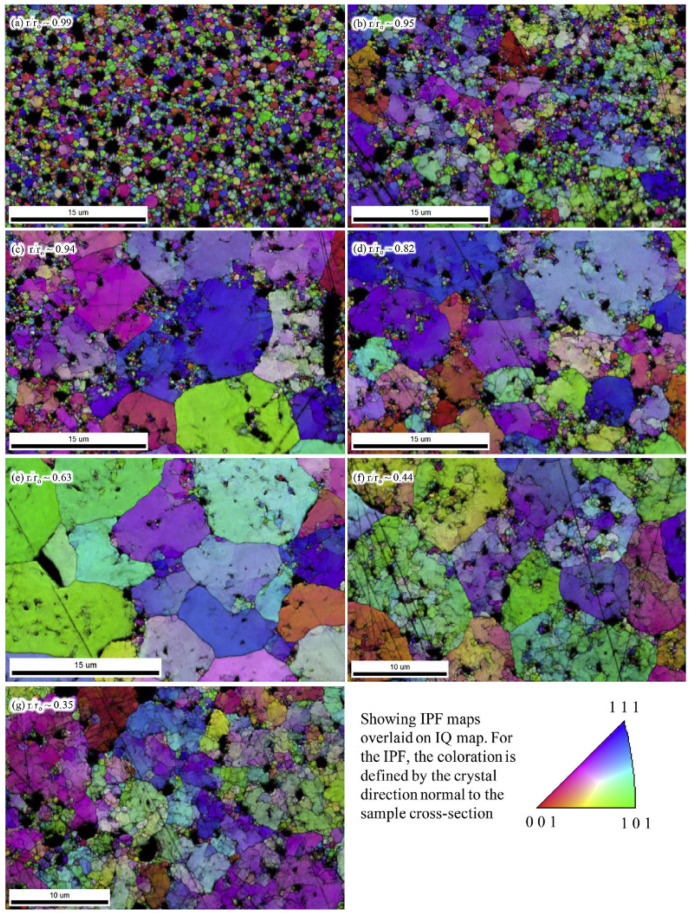
Locations of select EBSD scans moving from the fuel-cladding interface (r/r_o_~0.99) toward the fuel center r/r_o_~0.35, (**a**–**g**) [26].

Experimental characterization results support this hypothesis to some extent. Gerczak et al. [26] noted that, as shown in Figure 3, near the center of the fuel, only the original grains and newly formed subgrains with low-angle boundaries (typically associated with the polygonization mechanism) are observed. At a relative radius of 0.63, there is a noticeable reduction in subgrain formation, a relative decrease in porosity, and the appearance of new grains with high-angle boundaries (commonly considered to be formed through recrystallization). Additionally, recrystallized grains appear at the boundary points between the original grains and subgrains, further corroborating the aforementioned nucleation perspective. As one moves outward to what is generally recognized as the thickness of the HBS, approximately 0.01R from the edge, there is an increase in recrystallized grains and subgrain formations, along with a rise in porosity distribution.

EPMA characterization results [26] further support this perspective, revealing a clear threshold boundary for grains at the edge of the fuel. When the position’s energy exceeds a certain threshold (reflecting the combined effects of the aforementioned factors), there is a rapid increase in high-angle boundaries and a swift decrease in low-angle boundaries. This phenomenon may suggest that exceeding an energy threshold triggers the rapid onset of the recrystallization process.

**Figure 4:**
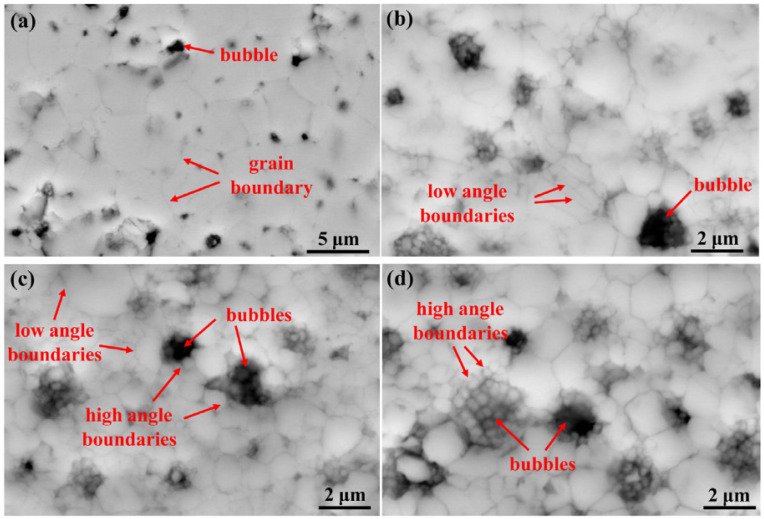
High magnification SEM micrographs illustrating the HBS with local burn-up of (**a**) 48 GWd/tU; (**b**) 87 GWd/tU; (**c**) 105 GWd/tU; and (**d**) 120 GWd/tU [29].

A comparative observation of the SEM images shown in Figure 4, as provided by Xiao et al. [29], clearly shows the sequential appearance of low-angle boundaries with increasing burn-up, where low-angle and high-angle boundaries coexist, and high-angle boundaries are densely packed around the bubbles. This structural relationship with burn-up supports the hypothesis of a hybrid mechanism.

Veshchunov [48] referenced the experimental results of Une et al. [11], which demonstrated that under relatively low burn-up conditions of 44 GWd/tU at a temperature of 1000 K, low-angle boundaries were present. In contrast, at a higher burn-up of 83 GWd/tU, high-angle boundaries were observed. This also suggests the possibility of a threshold that serves as a boundary for a hybrid control mechanism.

From a simulation perspective, there is a clear inclination toward exploring beyond a single mechanism. Jonnet [64] indicated that the future development of his polygonization-based model involves coupling it with Rest’s recrystallization model, as mentioned above, implying that polygonization acts as a precursor to the recrystallization process. D. Pizzocri et al. [50] also noted that recrystallization and polygonization mechanisms are essentially related. Abdoelatef’s model [53] treats grain refinement as a phase transformation without attempting to distinguish between recrystallization and polygonization principles. Although Baron et al. [69] considered polygonization to be the dominant mechanism, their energy assessments also partially support the hybrid mechanism viewpoint.

The experimental and simulation studies listed above indicate that a single control mechanism cannot fully explain the observed phenomena, indirectly supporting the proposal of a hybrid control mechanism.

## 4. Conclusions and Perspectives

### 4.1. Summary of Hybrid Mechanisms in HBS Formation

As described in the previous sections, it summarizes experimental observations related to the potential formation of HBS, the establishment of models based on these experiments, and the perspectives of various scholars. Starting from the two potential mechanisms for HBS formation—polygonization and recrystallization—and based on observations from experimental and simulation studies, it is proposed that dislocation evolution is a common feature between the two. It is further suggested that these mechanisms manifest differently under varying external conditions, reflecting distinct energy-based behaviors. Based on the proposed viewpoints, corresponding supporting experimental and simulation studies are listed, which, to some extent, substantiate the reasonableness of this hypothesis.

Regarding the hybrid control mechanism, it offers a better interpretation of the complete process from the emergence of low-angle boundary subgrains to the coexistence of recrystallized grains with high-angle boundaries and subgrains with low-angle boundaries, culminating in the significant appearance of pores primarily filled with recrystallized grains characterized by high-angle boundaries. This provides a theoretical foundation, as discussed in Section 3.2, for developing models to understand the microstructural evolution induced by changes in burn-up [26,29]. It also suggests the possibility of fully considering the impact of the dynamic microstructural evolution, which continuously changes with burn-up, on studies of fission gas behavior and nuclear fuels’ performance degradation.

Recent simulation studies have generally focused on simulating the formation of recrystallization cores in the HBS region and the subsequent recrystallization processes, with little attention given to the formation of subgrains before the formation of recrystallization cores. Experimental observations [26] indicate that the boundary locations of these substructures also serve as nucleation sites for recrystallized grains, and pore accumulation at these sites suggests a trapping effect for fission gases, which could further induce recrystallization processes. The lack of theoretical elucidation and model establishment regarding grain size variations at different locations in PWR fuel rods may hinder the understanding of fuel behavior under high burn-up conditions and impede efforts to push fuel toward higher burn-ups.

### 4.2. Recommendations for Future Modeling and Experimental Research

Regarding recommendations for modeling work, first, it is essential to model the complete grain evolution process across the entire fuel cross-section, clarifying the conditions under which subgrains will form. For instance, the attempts made by Baranov et al. [70] to analyze this phenomenon using dislocation dynamics provide insights for modeling the polygonization mechanism that leads to subgrain formation. Based on the aforementioned hybrid mechanism control, a reasonable set of energy thresholds should be designed to vary with burn-up and temperature, allowing these thresholds to adequately describe both the dominant mechanisms suggested by most current experimental data and the transition regions, thus clarifying and refining the energy-related hybrid mechanism theory.

Secondly, it is crucial to consider the behavior of fission gases directly impacted by grain size evolution. For commonly used diffusion equations, grain size often determines diffusion distance, typically modeled using constant or step-function forms for normal micron-sized grains and HBS nano-grains, as well as exponential fitting or mechanistic grain growth models [34,71,72]. There is a lack of continuous and comprehensive consideration in grain refinement modeling. Additionally, since dislocation effects have been incorporated into the research of Rest et al. [34,72], it is worth investigating whether the subgrains formed by dislocation wall rotation may have a unique impact on fission gas behavior, especially since such trapping effects have been observed experimentally. Restani [73] also noted that the pressure and release conditions of the HBS, regardless of whether they are below the threshold release temperature, need to be considered separately. It is also important to address how various behaviors transform within different regions, such as the transition from intragranular bubbles to grain boundary bubbles, and how to identify internal regions, HBS transition regions, and HBS regions. Adjusting the corresponding parameters based on these distinctions will be crucial for analyzing fission gas behavior in the context of higher burn-ups.

Finally, the implications of grain evolution and the various impacts of the HBS should be integrated into analytical programs. The presence of grain boundaries significantly reduces the thermal conductivity of fuel, but the impact on subgrains has not been adequately addressed, nor has the evolution of grain size been included in commonly used thermal conductivity calculations. Currently, there are some attempts to account for the effects of HBS on thermal conductivity. Xian-Ming Bai et al. [65] conducted an analytical model on the effects of different Xe concentrations, bubble porosities, and grain sizes on thermal conductivity, aiming to evaluate how HBS impacts the thermal conductivity of UO_2_. The larger porosity characteristic of HBS significantly reduces thermal conductivity and requires consideration of the effects during the depletion process. Gado et al. [69] considered the local changes caused by the formation of HBS in the FUROM code: the local porosity increases, which results in a decrease in the UO_2_ density. Young’s modulus is also notably affected by HBS, as Terrani et al. [9] have established a relationship between Young’s modulus and burn-up. Furthermore, beyond studying the influencing parameters, future research may explore modeling from the perspective of thermal-mechanical coupling or irradiation-thermal-mechanical coupling, with an emphasis on uniformization. Kulacsy [68] developed a model based on the assumption that during irradiation, the pores reach dislocation punching overpressure, and the increased overpressure due to rising temperature leads to fuel fragmentation. This model explains the fracturing of HBS in the fuel pellet during an LOCA and successfully simulates the transient release of fission gases and the depletion of gases in the matrix. Capps et al. [66] suggest that HBS exhibits lower yield stress compared to fresh fuel, and the model calculations indicate that the fracturing of high burn-up zone fuel is not caused by thermal stress under LOCA conditions. Currently, programs such as MFPR [48], TRANSURANUS [50], BISON [74], and DIONISIO [75] have partially considered the influence of HBS. In summary, how to effectively incorporate these macroscopic phenomena observed in experiments into fuel performance analysis programs will be a critical research area for high burn-up fuels under operational or accident conditions.

For recommendations regarding experimental work, with the continuous advancement of experimental analysis techniques, it may be necessary to conduct further experimental studies at higher burn-up points. For example, Veshchunov [48] noted that the burn-up primarily characterized by polygonization (44 GWd/tU) is lower than the burn-up required for observing recrystallization (83 GWd/tU). Additionally, such observations are generally limited by sample numbers, lacking a comprehensive characterization of microstructural features across different burn-ups or, more broadly, across the entire fuel cross-section (reflecting varying burn-ups and temperatures). This limitation somewhat restricts experimental observations regarding the formation mechanisms of HBS.

Moreover, with the development of auxiliary tools and interdisciplinary approaches, the formation of HBS can be explored through new methods. For example, research by Kinoshita et al. [17] indicates that certain chemical elements play a role, while Soba et al. [76] and Frazer [77] et al. argue that elemental migration and the formation of precipitates have a significant impact. The method [67] of using MATLAB tools to process SEM images to restore bubble morphology, quantity distribution, and interconnection can deepen our understanding of the effect of bubbles on HBS formation. The FIB-SEM 3D examination [78] can also provide similar insights. These new insights and tools from various fields will provide fresh perspectives on the formation of HBS. Existing studies suggest that HBS result from the local minimization of internal energy, driven by the need to adapt to the harsh irradiation–thermal–mechanical conditions within the reactors. This structure may not solely bring adverse effects during fuel operation. For instance, Spino et al. [79] suggest that nano-crystalline UO_2_ could serve as a potential high-performance fuel with gas retention capabilities and resistance to particulate cladding interactions. Its advantages regarding oxidation resistance have also been observed in studies by Baranov et al. [70], warranting further exploration. Multiscale experimental observations [80] are also essential for understanding the formation mechanisms of HBS.

Finally, the formation mechanism of HBS remains an open question, lacking decisive conclusions. The viewpoints presented in this paper are merely inferences drawn from the current body of research. The formation of HBS and their implications are unavoidable topics in extending the lifespan of commercial reactor fuels towards higher burn-up expectations, transcending mere academic discourse. Utilizing increasingly mature experimental characterization techniques and multi-scale coupled analysis methods, combined with perspectives from physics, chemistry, and materials science, will provide a reasonable solution to the challenge of HBS formation.

## Figures and Tables

**Table 1:** Formation conditions of HBS from the experimental perspective (blank indicates values not provided).

Temperature (°C)	Burn-Up (GWd/tU)	Original Grain Size (μm)	Reactor, Type	Supporting Views	Researcher (Ref.)
400	60–70		BWR, rod	recrystallization	Nogita/Une [10,11]
<800	70	17	PWR, rod	both	Spino [13]
	45 **	10	PWR, rod	polygonization	Noirot [14]
<1100	55–65			polygonization	Baron [2]
<1100 ± 100	55–82	9.7 ± 0.8	disk fuel	polygonization	T. Sonoda [17]
550 *	100 *	~8	disc samples	polygonization	Gerczak [26]
	220 ± 20 *		PWR, rod		Hiernaut [28]
300–400	87	~8	RR, disk fuel	both	Xiao [29]
~750	27		AGCR, rod		Baker [30]
<1200 *	76 *		LWR, rod		Cappia [31]
<1000 *	61	~9	PWR, rod	polygonization	Zacharie-Aubrun I [33]

Note: * according to the end-state data provided in the paper. ** average burn-up.

**Table 2:** Formation conditions of HBS from the numerical simulations (blank indicates values not provided).

Temperature (°C)	Burn-Up (GWd/tU)	Original Grain Size (μm)	Supporting Views	Researcher (Reference)
<727	~50	10~20	recrystallization	Rest [39,40]
<1027	55–82	~9	both	Veshchunov [48]
<1027–1127	100		polygonization	Xiao [49]
827	48.6		polygonization	Permann [58]
<1000	50	9–12	polygonization	D. Pizzocri [50]
927	70–75	2.9–4.1	polygonization	Abdoelatef [53,54]
927	45	6.4		K. Ahmed [55]
650	58	10	both	Amani Cheniour [57]
800	59			
827	64.2	10		Permann [58]
400	50	8.01	polygonization	Y. Jiang [60]
<1000	70			Jonnet [64,65,66]

## Data Availability

The data presented in this study are available on request from the corresponding authors.
